# Working memory after and during 6 Hz transcranial alternating current stimulation

**DOI:** 10.1192/j.eurpsy.2021.1303

**Published:** 2021-08-13

**Authors:** Y. Pavlov, D. Kasanov, O. Dorogina

**Affiliations:** 1 Department Of Psychology, Ural Federal University, Ekaterinburg, Russian Federation; 2 Institute Of Medical Psychology, University of Tuebingen, Tuebingen, Germany

**Keywords:** Working memory, EEG, tACS

## Abstract

**Introduction:**

Transcranial alternating current stimulation (tACS) is a non-invasive brain stimulation technique allowing to induce changes in oscillatory activity. Theta activity has been reported to play a major role in maintenance of information in working memory (WM).

**Objectives:**

The current study had the initial goal to check the effect of theta tACS on accuracy and resting state EEG in a set of match-to-sample WM tasks.

**Methods:**

In the first experiment, we tested 31 participants in the WM task after 20-min tACS applied at Fpz and CPz at 6 Hz, 1 mA.). In the second experiment, we compared the after-effects and online effects of the stimulation in a sample of 25 individuals. Five similar 25-min blocks filled with the same working memory task were distributed over 3 days. We assessed the same group of participants in all three sessions. On the Training day, the participants performed one block without stimulation. On the Sham-Verum day (SV), the first block with Sham stimulation followed by the second block with Verum stimulation. On the Verum-Sham day (VS), the blocks order reversed.

**Results:**

After-effects of the stimulation did not produce any significant changes either in behavior (accuracy in the task) or resting-state EEG (theta frequency band spectral power in the first experiment. In the second experiment, 6 Hz tACS delivered before the WM task was not able to produce any observable changes in working memory performance. The same hold true for online stimulation.

**Conclusions:**

Theta frequency tACS applied to Fpz-CPz electrodes is not an efficient method to improve WM.

